# Tight junction protein expression from peritoneal dialysis effluent

**DOI:** 10.1080/0886022X.2019.1686018

**Published:** 2019-11-14

**Authors:** Sua Kim, Eun Young Choi, Chor Ho Jo, Gheun-Ho Kim

**Affiliations:** a Institute of Biomedical Science, Hanyang University College of Medicine, Seoul, Korea;; b Department of Internal Medicine, Hanyang University College of Medicine, Seoul, Korea

**Keywords:** Claudin, immunoblotting, occludin, peritoneal dialysis, tight junction

## Abstract

**Background:** We hypothesized that tight junction (TJ) proteins may have a role in paracellular transport of solute and water in peritoneal dialysis (PD) patients. Previous studies on TJ proteins in PD patients have used only cultured human peritoneal mesothelial cells (HPMCs). This study was undertaken to test whether TJ proteins are directly identified from PD effluent and whether their expressions are associated with functional parameters of PD.

**Methods:** Dialysis effluents were collected from 40 patients undergoing PD, after the peritoneal equilibration test (PET). Different molecular sizes of Amicon Ultra-15 Centrifugal Filter Units were used to concentrate and purify proteins in PD effluents, and immunoblot analyses for occludin, ZO-1, and claudins were carried out to test for their existence and relationships with peritoneal clearance or results of the PET.

**Results:** Immunoblotting from PD effluents revealed discrete bands of occludin (∼65 kDa), ZO-1 (∼215 kDa), claudin-1 (∼22 kDa), and claudin-15 (∼22 kDa) in all 40 patients. The peritoneal creatinine clearance inversely correlated with the protein expression of claudin-1 (*r*= −0.369, *p*= .019), and the dialysate-to-plasma creatinine ratio at 4 h PET correlated with occludin (*r* = 0.396, *p*= .011) and inversely correlated with claudin-15 (*r*= −0.393, *p*= .012).

**Conclusion:** In PD patients, expression of peritoneal TJ proteins can be estimated from the dialysis effluent and may be used as novel peritoneal biomarkers.

## Introduction

Peritoneal dialysis (PD) is possible through unidirectional transport from the vascular space to the peritoneal fluid *via* the peritoneal membrane, which consists of the capillary wall, interstitium, and mesothelium [1]. However, molecular mechanisms of peritoneal transport remain elusive, although aquaporin-1 at the endothelium of the capillary wall is known to play a role in regulating water permeability of the peritoneal membrane [2]. Here, we focused on the mesothelium and hypothesized that TJ proteins may have a role in paracellular transport of solute and/or water in PD patients. Previous studies on TJ proteins in PD patients have used only cultured human peritoneal mesothelial cells (HPMCs) [3–6]. We hypothesized that TJ proteins can be directly identified from PD effluent and may reflect peritoneal transport characteristics such as peritoneal clearance and parameters measured during the peritoneal equilibration test (PET). This study was undertaken to explore the expression of TJ proteins from PD effluent and to investigate their relationships with functional parameters of PD.

## Materials and methods

**Figure 1.**
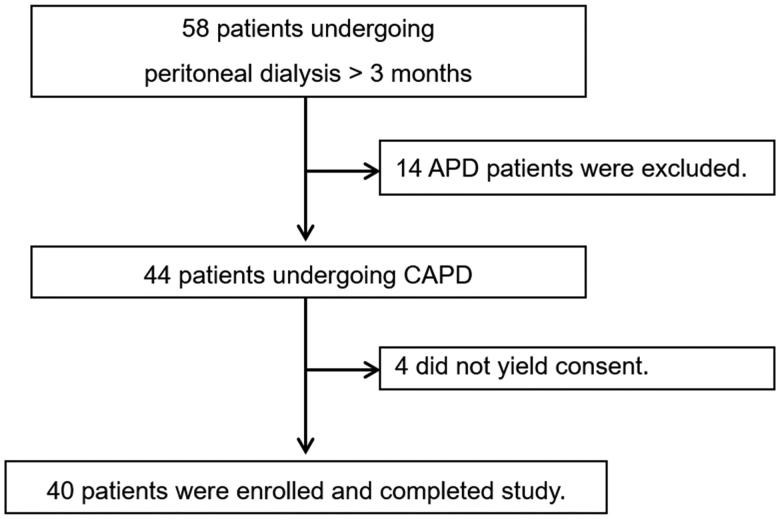
Flow chart of patient enrollment. APD: automated peritoneal dialysis; CAPD: continuous ambulatory peritoneal dialysis.

A total of 58 patients undergoing PD were identified in our hospital. We excluded 14 patients who were receiving automated PD, and we could not obtain consent from 4 patients. Finally, 40 patients undergoing continuous ambulatory PD were enrolled and completed study (Figure 1).

The following performance data of PD were obtained from 24-h collections of dialysate and urine: peritoneal dialysate inflow and outflow, daily ultrafiltration, peritoneal and renal KT/V urea, peritoneal and renal creatinine clearance, and residual renal function. PET was done using a 4.25% glucose solution over 4 h, and dialysate/plasma ratios of creatinine, glucose, and sodium were measured at baseline, 1 and 4 h [7]. To concentrate various TJ proteins in PD effluents, different molecular sizes (3, 30, and 100 kDa) of Amicon Ultra-15 Centrifugal Filter Units (Millipore, Bedford, MA) were used before preparing samples for immunoblotting.

**Figure 2.**
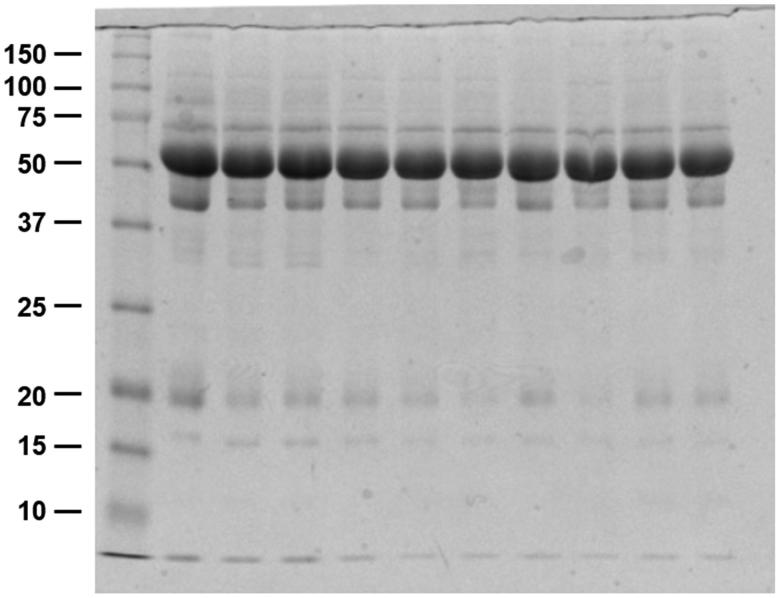
Coomassie blue-stained SDS/12% polyacrylamide gel from peritoneal dialysis effluents. Each lane was loaded with a protein sample from a different patient, and a strong band density is noted at ∼65 kDa in all patients undergoing peritoneal dialysis.

Equal amounts of protein were electrophoresed on sodium dodecyl sulfate-polyacrylamide gels and transferred onto nitrocellulose membranes. Membranes were probed overnight at 4 °C with primary antibodies: mouse monoclonal anti-claudin-1, mouse monoclonal anti-claudin-2, rabbit polyclonal anti-claudin-4, monoclonal anti-claudin-7, polyclonal anti-claudin-8, polyclonal anti-claudin-15, rabbit polyclonal anti-ZO-1 (Invitrogen, Carlsbad, CA), rabbit polyclonal anti-occludin (Thermo Fisher, Rockford, IL), and monoclonal anti-β-actin, (Sigma, St. Louis, MO). Secondary antibodies were goat anti-rabbit or goat anti-mouse IgG conjugated to horseradish peroxidase (Jackson ImmunoResearch, West Grove, PA). The sites of antibody–antigen reactions were viewed using enhanced chemiluminescence (WEST-ZOL^ⓡ^Plus, Intron Biotechnology, Seongnam, Korea), and the band densities on immunoblots were quantified by densitometry using a laser scanner and Quantity One software version 5.2 (Bio-Rad, Hercules, CA). For quantitative comparisons, the albumin band detected by the Coomassie blue staining was used as a loading control (Figure 2). At the same time, clinical data were collected in patient demographic characteristics, fundamental laboratory findings, residual renal function, peritoneal clearance, and results of PET. The study protocol was approved by the Institutional Review Board of Hanyang University (No. 2015-05-028).

Data are expressed as mean ± standard deviation (SD) or frequency (proportion). Statistical comparisons between groups were performed using the Mann–Whitney *U* test, and correlations between variables of interest were analyzed by linear regression (Abacus Concepts, Berkeley, CA). *p*< .05 was considered to indicate statistical significance.

## Results

The patient group consisted of 23 men (57.5%) and 17 women (42.5%) with a mean age of 54.1 ± 15.6 years. The proportion of diabetic patients was 20%. The mean duration of PD was 41 months, ranging from 5 to 141 months. The performance data of PD and the results of PET are shown in Tables 1 and 2, respectively. The latter revealed that four patients had low, 18 had low average, 17 had high average, and one had high peritoneal transport characteristics.

**Table 1.** Performance data of peritoneal dialysis (*n* = 40).

Parameters	Value
Peritoneal dialysate inflow (mL/d)	7525 ± 913
Peritoneal dialysate outflow (mL/d)	8461 ± 1245
Net ultrafiltration (mL/d)	936 ± 597
Peritoneal KT/V urea	1.749 ± 0.300
Renal KT/V urea	0.239 ± 0.350
Total KT/V urea	1.988 ± 0.472
Peritoneal creatinine clearance (L/week)	42.17 ± 7.61
Renal creatinine clearance (L/week)	13.96 ± 20.86
Total creatinine clearance (L/week)	56.39 ± 22.02
Residual renal function (mL/min)	1.091 ± 1.603
nPNA (g/d)	1.037 ± 0.229

nPNA: normalized protein nitrogen appearance.

Values are mean ± SD.

**Table 2.** Results of peritoneal equilibration test (*n* = 40).

Parameters	Value
Creatinine D1/P1 ratio	0.401 ± 0.095
Creatinine D4/P4 ratio	0.680 ± 0.102
Glucose D0/P0 ratio	35.41 ± 8.73
Glucose D1/P1 ratio	14.62 ± 5.44
Glucose D4/P4 ratio	8.932 ± 2.922
Sodium D0/P0 ratio	0.953 ± 0.037
Sodium D1/P1 ratio	0.895 ± 0.027
Sodium D4/P4 ratio	0.917 ± 0.028
Glucose D1/D0 ratio	0.595 ± 0.056
Glucose D4/D0 ratio	0.298 ± 0.051

D0: dialysate at baseline; D1: dialysate at 1 h; D4: dialysate at 4 h; P0: serum at baseline; P1: serum at 1 h; P4: serum at 4 h.

Values are mean ± SD.

**Figure 3.**
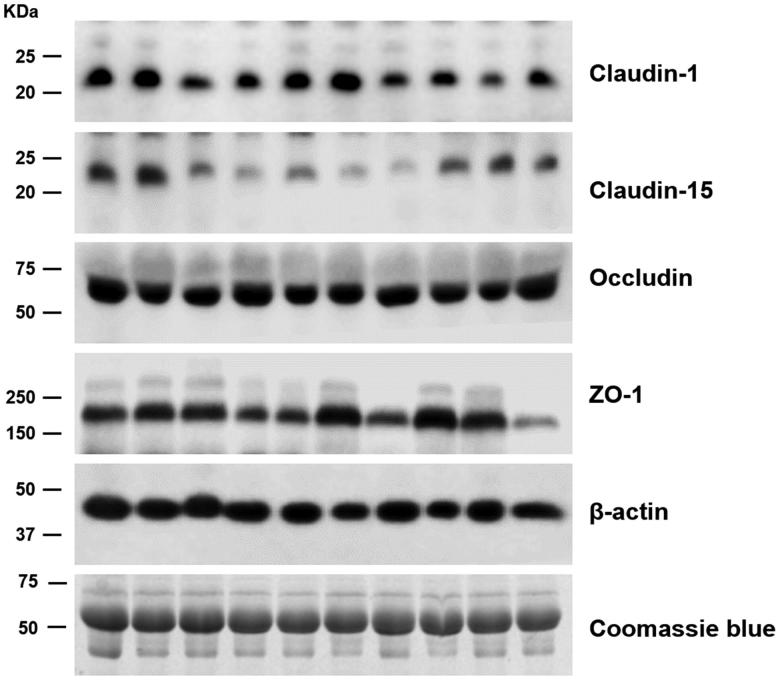
Immunoblots of peritoneal dialysis effluents probed with tight junction protein-specific antibodies. The immunoblots were reacted with anti-claudin-1 (∼22 kDa band), anti-claudin-15 (∼22 kDa band), anti-occludin (∼65 kDa band), and anti-ZO-1 (∼215 kDa band). Each lane was loaded with a protein sample from a different patient, and representative immunoblots are shown from 10 patients undergoing peritoneal dialysis.

Immunoblotting from PD effluents revealed discrete bands of occludin (∼65 kDa), ZO-1 (∼215 kDa), claudin-1 (∼22 kDa), and claudin-15 (∼22 kDa) in all 40 patients. However, claudin-2, claudin-4, claudin-7, and claudin-8 were not detected. Representative immunoblots of claudin-1, claudin-15, occludin, and ZO-1 are shown in Figure 3.

**Figure 4.**
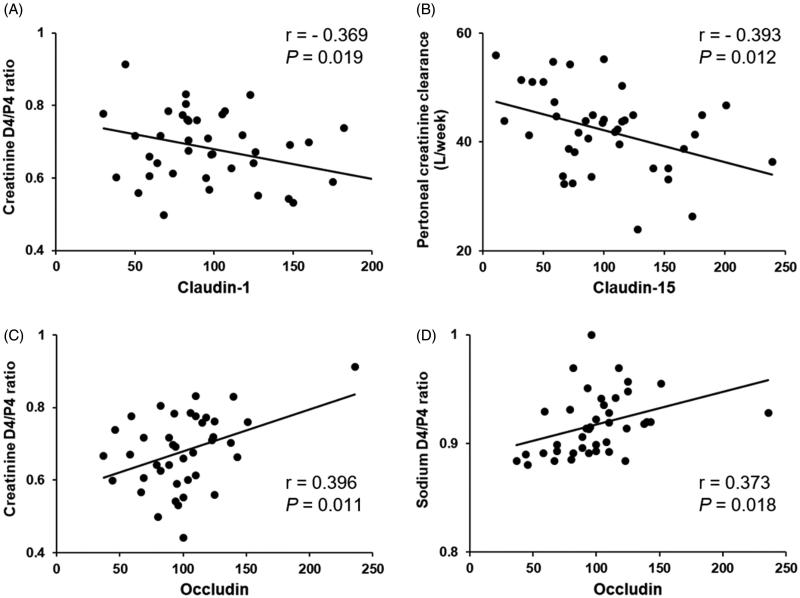
Relationships between the expression level of TJ proteins and peritoneal transport characteristics. (A) Claudin-1 inversely correlated with the dialysate-to-plasma creatinine ratio at 4 h during the peritoneal equilibration test (PET). (B) Claudin-15 inversely correlated with peritoneal creatinine clearance. (C) Occludin correlated with the dialysate-to-plasma creatinine ratio at 4 h PET. (D) Occludin correlated with the dialysate-to-plasma sodium ratio at 4 h PET.

To examine the functional significance of TJ protein expression from PD effluents, we tested if the expression level of TJ proteins was linked to peritoneal transport characteristics. Figure 4 shows that claudin-1 inversely correlated with the dialysate-to-plasma creatinine ratio at 4 h during PET (*r*= −0.369, *p*= .019) and that claudin-15 inversely correlated with peritoneal creatinine clearance (*r*= −0.393, *p*= .012). Furthermore, the protein level of occludin correlated with the dialysate-to-plasma creatinine ratio (*r* = 0.396, *p*= .011) and the dialysate-to-plasma sodium ratio (*r*=.373, *p*= .018) at 4 h during PET. We found no significant correlations between PD duration and TJ protein expressions from PD effluent (claudin-1, *p*= .189; claudin-15, *p*= .464; occludin, *p*= .528; ZO-1, *p*= .305).

## Discussion

To the best of our knowledge, this study showed for the first time that expression of peritoneal TJ proteins can be estimated from PD effluent. Previously, cultured mesothelial cells from human [3–6] or rat [8] omenta were used to investigate expression of peritoneal TJ proteins. However, there should be a difference between *in vivo* and *in vitro* expression of peritoneal TJ proteins because the *in vitro* culture conditions may modify protein expressions. We developed a novel and clinically applicable method of estimating peritoneal TJ protein expression from PD patients. Because PD effluent is too dilute for measuring TJ proteins, centrifugal filter devices were successfully used for concentrating and purifying the peritoneal fluid samples [9]. However, concentration or purification procedures from PD effluent might favor biases in quantifying TJ proteins.

According to Retana et al. claudin-1, -2, and -8; ZO-1; and occludin were localized in cultured HPMCs [6]. Among these, we could detect claudin-1, ZO-1, and occludin from PD effluents. Caludin-2 and -8 may be more difficult to detect due to their intranuclear localization [6]. Claudin-15 was also identified from our PD patients, suggestive of a role in transport of ions and/or water through the peritoneum. Its deficiency in mice was reported to decrease the number of TJ strands and produce megaintestine [10].

This study showed that the expression levels of claudin-1, claudin-15, and occludin protein weakly but significantly correlated with peritoneal transport characteristics. In particular, the inverse association between claudin-15 and peritoneal creatinine clearance may be compatible with the potential role of claudin-15 in paracellular barrier formation [10]. We believe that TJ proteins in PD effluent may reflect the peritoneal membrane activities of the patients. Lack of correlations between TJ protein expression levels and PD duration was also reported by Retana et al., using cultured HPMCs s from PD patients [11]. However, we cannot but admit that our results are too limited to define the role of individual TJ protein in peritoneal membrane function.

## Conclusion

In PD patients, expression of peritoneal TJ proteins can be estimated from the dialysis effluent and may be used as novel peritoneal biomarkers. This approach is practically valuable because previous studies on peritoneal TJ proteins were only from cultured HPMCs. Further studies are required to elucidate the role of TJ proteins in peritoneal transport and dialysis.
